# Dose Correlation of Danggui and Chuanxiong Drug Pairs in the Chinese Medicine Prescription Based on the Copula Function

**DOI:** 10.1155/2020/2372746

**Published:** 2020-11-20

**Authors:** Xuan Zhao, Wei Lin, Jiawei Li, Yunhui Chen, Anamica Patel, Hailiang Zhao, Guoxin Han, Yiwen Hao, Chaomei Fu, Zejuan Huang, Mingyue Zheng, Peng Hu

**Affiliations:** ^1^School of Pharmacy, Chengdu University of Traditional Chinese Medicine, Chengdu 611137, China; ^2^School of Management, Chengdu University of Traditional Chinese Medicine, Chengdu 611137, China; ^3^College of Clinical Medicine, Chengdu University of Traditional Chinese Medicine, Chengdu 611137, China; ^4^Observatory Evidence Service, Public Health Wales, Cardiff CF10 4BZ, UK; ^5^Adelaide Medical School, University of Adelaide, Adelaide, SA 5005, Australia; ^6^School of Health and Rehabilitation, Chengdu University of Traditional Chinese Medicine, Chengdu 611137, China

## Abstract

Dosage is essential for studying the compatibility and effectiveness of traditional Chinese medicine. Danggui and Chuanxiong are widely used in traditional Chinese medicine for ailments and treatment of various disorders. 628 traditional Chinese medicine prescriptions containing Danggui and Chuanxiong were extracted from the self-built prescription database and screened for the three groups of prescriptions, i.e., irregular menstruation, sores, and stroke. We processed and tested the dosage of Danggui and Chuanxiong and selected the optimal copula function, Gumbel copula function, from the Archimedes function family and elliptical copula function family to establish the data model. To establish the presence of a correlation between the dose of Danggui and Chuanxiong, a graph of the joint distribution function of rank correlation coefficients, Kendall's rank correlation coefficient and Spearman's rank correlation coefficient, was used. Our results suggest that the model using the Gumbel copula function better reflects the correlation between the dose of Danggui and Chuanxiong. For irregular menstruation, sores, and strokes, Kendall's rank correlation coefficients were 0.6724, 0.5930, and 0.7757, respectively, and Spearman's correlation coefficients were 0.8536, 0.7812, and 0.9285, respectively. In all three prescription groups, the dose of Danggui and Chuanxiong was positively correlated, implying that, as the dosage of one drug increases, the dosage of the other increases as well. From the perspective of data mining and mathematical statistics, the use of the copula function model to evaluate the correlation between the prescribed dosage of the two drugs was innovative and provided a new model for the scientific interpretation of the compatibility of traditional drugs. This might also serve to guide the clinical use of traditional Chinese medicine.

## 1. Introduction

Combining drugs has been a common practice in clinical research and application throughout history. Two such drugs Radix *Angelica sinensis* (Danggui) and *Rhizoma Ligustici Chuanxiong* (Chuanxiong) were initially recorded in Shennong's Herbal Classic in traditional Chinese medicine (TCM). Danggui is the root of the umbelliferous plant *Angelica* [1]. It has the effects of promoting blood circulation, regulating menstruation, and relieving pain and is often used for women with irregular menstruation and dysmenorrhea. Chuanxiong is the dired rhizome of an umbelliferous plant (*Rhizoma Ligustici Chuanxiong*), which promotes blood circulation and Qi and helps relieve pain [2]. Combining these two drugs has been a widespread practice in TCM and has been recorded throughout history, for example, use of Siwu Tang (Danggui, Chuanxiong, Baishao, white peony root, and *Rehmannia glutinosa*) and Shenghua Decoction (Danggui, Chuanxiong, peach kernel, dried ginger, and licorice). While the former was prescribed in Heyaofangfang to treat irregular menstruation during the Song dynasty, the latter was used to treat postpartum lochia and lower abdominal pain during the Ming dynasty. Danggui and Chuanxiong drug pair is used in internal medicine, gynecology, and surgery [3, 4]. Various pharmacological studies have shown this drug pair to be effective in regulating peripheral blood circulation, enhancing immunity, improving blood circulation, inhibiting uterine contraction, inhibiting platelet aggregation, antioxidation, antithrombosis, etc [3–5]. Most recently, Xionggui Decoction (again a combination of Danggui and Chuanxiong) has been developed as the Shunaoxin Dripping Pill to treat ischemic stroke [6].

**Figure 1:**
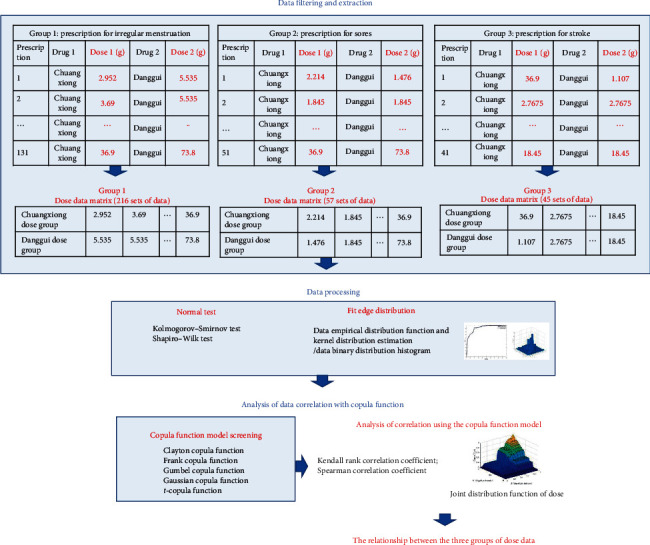
The diagram of analyzing the dose dependence of Danggui-Chuanxiong based on the copula function.

In TCM, compatibility of the drug pair and dose prescription are essential for effective treatment as different dose combinations of the same paired drugs can lead to different results [5, 6]. Danggui and Chuanxiong drug pairs have been commonly used by TCM doctors across generations [3, 4]; however, the dosage of different prescriptions is not specified. Additionally, previous studies on Danggui and Chuanxiong drug pairs have focused on the changes in their chemical composition, absorption metabolism, or pharmacological efficacy of single drugs and drug pairs [3–7]. In the absence of evidence regarding the compatibility of this drug pair, it is difficult to explain the correlation between Danggui and Chuanxiong in dose prescription. Combining data mining and mathematical statistics with pharmacology databases/library provides an opportunity for in-depth analysis of these drug pairs [7, 8], especially use of copula-based models [9] which have been validated in other medical studies [10, 11]. Therefore, this study aimed to explore the dose of Danggui and Chuanxiong drug pairs from the perspective of data mining and mathematical statistics by collecting prescriptions containing Danggui and Chuanxiong from the self-built prescription database. The doses of Danggui and Chuanxiong prescriptions in the three high-prevalent indications, irregular menstruation, sores, and stroke, were mathematically described [6, 12, 13], and copula function model analysis [14] was established to explore the correlation between Danggui and Chuanxiong doses used to treat these three conditions (Figure 1).

## 2. Materials and Methods

### 2.1. Data Screening

We collected 51,083 prescriptions (entire database) based on the recipes from the Han, Tang, Song, Ming, and Qing dynasties. Then, we screened the prescriptions containing Danggui and Chuanxiong drug pairs from the prescription database. We applied two main data collection standards: (1) we included only prescriptions that contained Danggui and Chuanxiong drug pairs and (2) as there are too many different doses, we only included the drug pair data for decoction prescription [15].

### 2.2. Database Establishment

A total of 628 prescriptions were included, and the dose database for Danggui and Chuanxiong prescriptions was created using Excel. Information for six variables was extracted: number, prescription name, dynasty, Danggui dose, Chuanxiong dose, and indicators. The methods used to measure medicines have changed over the years, such as using grams instead of the traditional weighing system in China. The conversion was different between the measurement unit in different years and the modern measurement unit. Therefore, the measurements used in the database might be different to the original measures used. We converted the dose to the drug according to the conversion principle [16] (Table 1) and examples of the Danggui and Chuanxiong database (Table 2).

We identified and recorded a total of 628 prescriptions containing Danggui and Chuanxiong and corresponding 16 indications (i.e., irregular menstruation, sores, woman futility, stroke, postpartum irritability, and fetal movement) for corresponding treatments. According to the classification of prescription data, there were 216 prescriptions for treating irregular menstruation, 57 prescriptions were used to treat sores, and 45 prescriptions were used to treat strokes. To ensure accuracy of the correlations, we only included the top three groups of prescriptions for irregular menstruation, sores, and stroke in this study.

### 2.3. Data Analysis

We calculated descriptive statistics (median and 95% confidence intervals (CI)) and conducted Kolmogorov–Smirnov test [17] or Shapiro–Wilk test [18] to test the distribution of the data. We used Ksdensity function to fit the marginal distribution of the data.

### 2.4. Analysis of Drug-Dose Correlation Using the Copula Function

#### 2.4.1. Archimedes Copula Function Family

We used the optimal copula function from the Archimedes function family (Gumbel copula function, Clayton copula function, and Frank copula function) and elliptical copula function family (Gaussian copula function and *t*-copula function) [19] and calculated the dose relationship between Danggui and Chuanxiong. We used the squared Euclidean distance algorithm to find the squared Euclidean distance between various copula functions and the empirical distribution function. The optimal copula function selected under this standard is the copula function with the shortest distance between the corresponding empirical copula functions. The distribution of the Archimedes copula function is [20]

(1) $$\begin{array}{c} C\left(u_{1},u_{2},\ldots ,u_{N}\right)=\left\{\begin{array}{cc} \varphi^{-1}\left[\varphi \left(u_{1}\right),\varphi \left(u_{2}\right),\ldots ,\varphi \left(u_{N}\right)\right], & \overset{N}{\underset{i=1}{\sum }}\varphi \left(u_{i}\right)\leq \varphi \left(0\right),\\ 0, & \text{otherwise,} \end{array}\right. \end{array}$$

where *φ*(*u*_*i*_) is the generator of the Archimedes copula function, *u* ∈ [0,1].

The expression of the binary Gumbel copula function is [9]

(2) $$\begin{array}{c} C\left(u,v\right)=\exp\left({\left(-{\left(-\ln u\right)}^{\alpha }+-{\left(-\ln v\right)}^{\alpha }\right)}^{1/\alpha }\right), \end{array}$$

where *α* is a parameter, which is more sensitive to the change of the upper tail. As *α* gradually increases, if the characteristic of the upper tail change is more obvious, the correlation between the variables is stronger.

The expression of the binary Clayton copula function is [19]

(3) $$\begin{array}{c} C\left(u,v\right)=\max\left({\left({\left(u\right)}^{-\alpha }+{\left(v\right)}^{-\alpha }-1\right)}^{-\left(1/\alpha \right)},0\right), \end{array}$$

where *α* is a parameter that is more sensitive to the changes of the lower tail. As *α* gradually increases, if the characteristic of the lower tail change is more obvious, the correlation between the variables is stronger.

The expression of the binary Frank copula function is [20]

(4) $$\begin{array}{c} C\left(u,v\right)=-\frac{1}{\alpha }\ln\left(1+\frac{\left(e^{-\alpha u}-1\right)\left(e^{-\alpha v}-1\right)}{e^{-\alpha }-1}\right), \end{array}$$

where *α* is a parameter, and its tail characteristics have symmetry, so it cannot capture the asymmetric tail correlation between variables.

#### 2.4.2. Elliptical Copula Family

The expression of the binary Gaussian copula function is [21]

(5) $$\begin{array}{c} C\left(u,v,\rho \right)=\int_{-\infty }^{\Phi^{-1}\left(u\right)}\int_{-\infty }^{\Phi^{-1}\left(v\right)}\frac{1}{2\pi \sqrt{1-\rho^{2}}}\exp\left(\frac{-\left(r^{2}+s^{2}-2\rho rs\right)}{2\pi \left(1-\rho^{2}\right)}\right)\text{d}r\text{d}s, \end{array}$$

where Φ^−1^(*u*) and Φ^−1^(*v*) are the inverse functions of the standard normal distribution function of Φ(*i*) and Φ(*v*) and *ρ* is the linear correlation coefficient of Φ^−1^(*u*) and Φ^−1^(*v*), and *ρ* ∈ (−1, 1).

The expression of the binary *t*-copula function is [21]

(6) $$\begin{array}{c} C\left(u_{1},u_{2};\rho ,v\right)=\int_{-\infty }^{\Gamma_{v}^{-1}\left(u_{1}\right)}\int_{-\infty }^{\Gamma_{v}^{-1}\left(u_{2}\right)}\frac{1}{2\pi \sqrt{1-\rho^{2}}}\left(1+\frac{s^{2}+t^{2}-2\rho st}{v\left(1-\rho^{2}\right)}\right)\text{d}s\text{d}t, \end{array}$$

where Γ_*v*_^−1^(*u*_1_)andΓ_*v*_^−1^(*u*_2_) are the inverse functions of the unary *t*-distribution functions of Γ_*υ*_(*u*_1_) and Γ_*υ*_(*u*_2_) and *ρ* is the linear correlation coefficient of Γ_*v*_^−1^(*u*_1_)andΓ_*v*_^−1^(*u*_2_), and*ρ* ∈ (−1,1).

#### 2.4.3. Correlation Measure

The correlation coefficient describes the degree of dependence between two variables and provides the core basis for accurate modelling [21]. The commonly used measures for the copula function are Kendall rank correlation coefficient and Spearman rank correlation coefficient. A range of correlation coefficients (from −1 to 0) implies a negative correlation between the variables, a correlation coefficient value of 0 implies that the variables are not related, and a range of correlation coefficients from 0 to 1 implies a positive correlation between the variables. Therefore, the closer the correlation coefficient value is to 1, the closer the correlation between the variables is [22].

If the marginal distributions of random variables *X* and *Y* are *F*(*x*) and *G*(*y*), respectively, and the corresponding copula function is *C*(*u*, *v*), where *u* = *F*(*x*), *v* = *G*(*y*), and *u*, *v* ∈ [0, 1], then the following two equations can be derived.

The expression of Kendall rank correlation coefficient *τ* is

(7) $$\begin{array}{c} \tau =4\int_{0}^{1}\int_{0}^{1}C\left(u,v\right)\text{d}C\left(u,v\right)-1. \end{array}$$

The expression of Spearman rank correlation coefficient *r* is

(8) $$\begin{array}{c} r=12\int_{0}^{1}\int_{0}^{1}C\left(u,v\right)\text{d}uv-3, \end{array}$$

where *C*(*u*, *v*) is the copula function of *u* and *v*.

## 3. Results

### 3.1. Descriptive Statistics

We calculated descriptive statistics for the Chuanxiong dose and Danggui dose in the three groups of prescriptions with irregular menstruation, sores, and stroke (Table 3).

### 3.2. Data Normality Test Results

We used Kolmogorov–Smirnov test and Shapiro–Wilk test to test the data for normal distribution (Table 4). We found the data were not normally distributed as the *P* values for both Kolmogorov–Smirnov test and Shapiro–Wilk test corresponding to the three indicators were less than the statistical significance level of 0.05.

### 3.3. Results of Data Fitting Marginal Distribution

**Figure 2:**
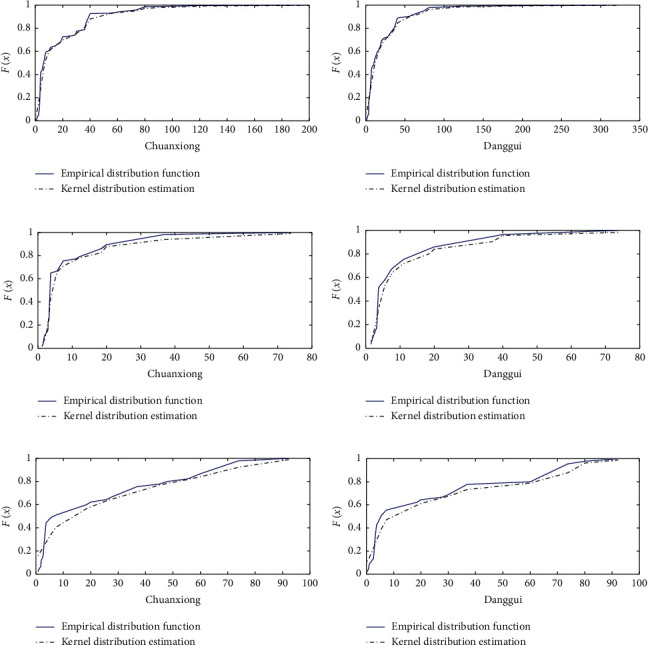
Empirical distribution function and kernel distribution estimation of drug-dose data. Note: the blue solid line represents the empirical distribution function; the black dotted line represents the kernel distribution estimation function; (a, c, and f) empirical distribution function and kernel distribution estimation of Chuanxiong; (b, d, and g) empirical distribution function and kernel distribution estimation of Danggui. (a) Chuanxiong (treatment of irregular menstruation). (b) Danggui (treatment of irregular menstruation). (c) Chuanxiong (treatment of sores). (d) Danggui (treatment of sores). (e) Chuanxiong (treatment of stroke). (f) Danggui (treatment of stroke).

**Figure 3:**
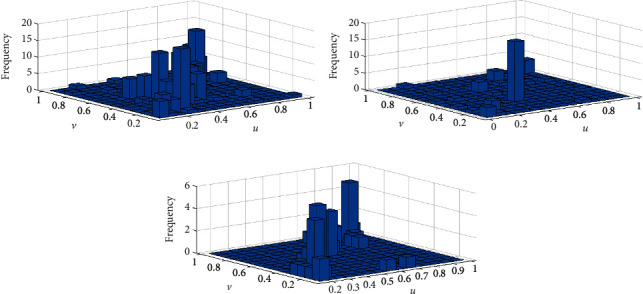
Histogram of frequency distribution. (a) Chuanxiong (U)/Danggui (V) of gynecology. (b) Chuanxiong (U)/Danggui (V) of sores. (c) Chuanxiong (U)/Danggui (V) of stroke.

**Figure 4:**
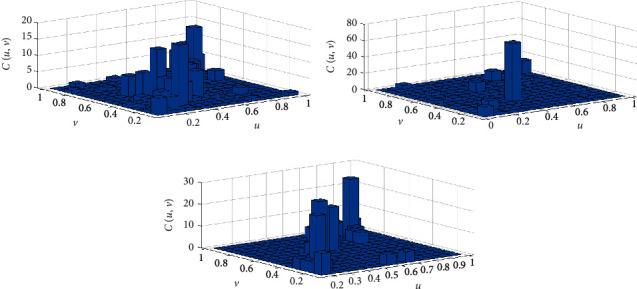
Histogram of probability distribution. (a) Chuanxiong (*u*)/Danggui (*v*) of gynecology. (b) Chuanxiong (*u*)/Danggui (*v*) of sores. (c) Chuanxiong (*u*)/Danggui (*v*) of stroke.

We used the Ksdensity function to calculate the marginal distribution of Danggui and Chuanxiong. The kernel distribution function helps avoid the error caused by the continuity and smoothness of the empirical distribution function, and this was used to fit the empirical distribution function [23]. Our results indicated that the nuclear distribution function and empirical distribution function of Danggui and Chuanxiong doses in the three prescription groups fit well (Figure 2). By looking at the tails of the binary distribution histograms of Danggui and Chuanxiong, it was determined the three prescription indicators share a strong correlation. Therefore, the copula function can be used to describe the nonlinear relationship between Danggui and Chuanxiong (Figures 3 and 4).

### 3.4. Copula Function Family Analysis of the Dose Correlation Results of Danggui and Chuanxiong

#### 3.4.1. Irregular Menstruation Group

The 216 prescriptions of the Danggui and Chuanxiong dose under this prescription group were substituted into the copula function, and the corresponding squared Euclidean distance and parameter estimates are calculated (Table 5).

Our results show that the Gumbel copula function is optimal to test the relationship between Danggui and Chuanxiong as the squared Euclidean distance of the Gumbel copula function is 0.0413, and the parameters are estimated to be 3.0522, which is the minimum value, using the following expression:

(9) $$\begin{array}{c} C\left(u,v\right)=\exp\left({\left(-{\left(-\ln u\right)}^{3.0522}+-{\left(-\ln v\right)}^{3.0522}\right)}^{1/3.0522}\right). \end{array}$$

**Figure 5:**
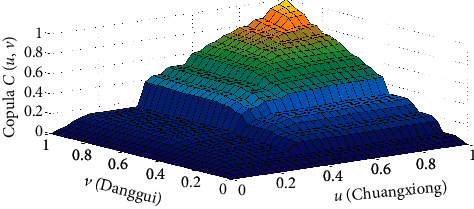
Joint distribution function of Danggui and Chuanxiong doses of irregular menstruation.

Here, copula *C*(*u*, *v*) is the joint distribution function of Danggui and Chuanxiong drawn according to the Gumbel copula, where the marginal distribution function of Chuanxiong is *U* (Chuanxiong) and the marginal distribution function of Danggui is V (Danggui) (Figure 5).

Based on the Gumbel copula function, we established a data model to describe the joint distribution function graph. The tail of the density function in the graph had a thicker morphological feature, which identified the correlation between Danggui and Chuanxiong. We estimated the Kendall rank correlation coefficient to be 0.6724 and the Spearman correlation coefficient to be 0.8536, indicating that the Danggui and Chuanxiong doses have a strong nonlinear positive correlation. This implies that when the dose of Danggui increases, the dose of Chuanxiong also increases significantly.

#### 3.4.2. Sores Group

The 57 prescriptions of Danggui and Chuanxiong doses were substituted into the copula function under this group, and the corresponding squared Euclidean distance and parameter estimates were measured (Table 6). Since the minimum squared Euclidean distance of the Gumbel copula function is 0.0077, the optimal function is the Gumbel copula function.

Our results show that the Gumbel copula function is optimal to test the relationship between Danggui and Chuanxiong as the squared Euclidean distance of the Gumbel copula function is 0.0193, and the parameters are estimated to be 2.4568 using the following expression:

(10) $$\begin{array}{c} C\left(u,v\right)=\exp\left({\left(-{\left(-\ln u\right)}^{2.4568}+-{\left(-\ln v\right)}^{2.4568}\right)}^{1/2.4568}\right). \end{array}$$

**Figure 6:**
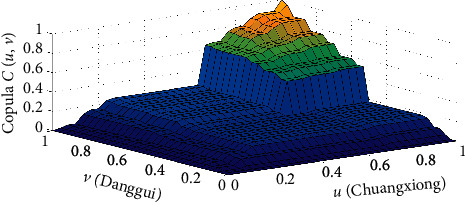
Joint distribution function of Danggui and Chuanxiong doses of sores.

Here, copula *C*(*u*, *v*) is the joint distribution function of Chuanxiong and Danggui drawn according to the Gumbel copula, where the marginal distribution function of Chuanxiong is *u* (Chuanxiong) and the marginal distribution function of Danggui is *v* (Danggui) (Figure 6).

We established a data model using the Gumbel copula function to describe the joint distribution function graph. We found this model to be a better reflection of the correlation between doses of Danggui and Chuanxiong. We estimated the Kendall rank correlation coefficient to be 0.5930 and Spearman correlation coefficient to be 0.7812, indicating a strong nonlinear positive correlation between the doses of Danggui and Chuanxiong. This implies that when the dose of Danggui increases, the dose of Chuanxiong will also increase significantly.

#### 3.4.3. Stroke Group

The 45 prescriptions of Danggui and Chuanxiong doses were substituted into the copula function under this group, and the corresponding squared Euclidean distance and parameter estimates were measured (Table 7).

Our results show that the Gumbel copula function is optimal to test the relationship between Danggui and Chuanxiong as the squared Euclidean distance of the Gumbel copula function is 0.0099, and the parameter estimation is 4.4580 using the following expression:

(11) $$\begin{array}{c} C\left(u,v\right)=\exp\left({\left(-{\left(-\ln u\right)}^{4.4580}+-{\left(-\ln v\right)}^{4.4580}\right)}^{1/4.4580}\right). \end{array}$$

**Figure 7:**
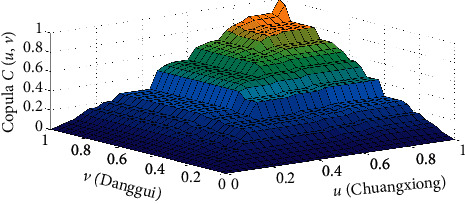
Joint distribution function of Chuanxiong and Danggui doses of stroke.

Here, copula *C*(*u*, *v*) is the joint distribution function of Danggui and Chuanxiong drawn according to the Gumbel copula, where the marginal distribution function of Chuanxiong is U (Chuanxiong) and the marginal distribution function of Danggui is V (Danggui) (Figure 7).

According to the Gumbel copula function, a data model was established to describe the joint distribution function graph. The tail of the density function in the graph has a thicker morphological feature, which can better capture the tail correlation feature between Danggui and Chuanxiong. It shows that the model of Danggui and Chuanxiong established by the Gumbel copula function can better reflect the correlation between Danggui and Chuanxiong. The Kendall rank correlation coefficient sum was 0.7757, and the Spearman correlation coefficient was 0.9285, indicating that the Danggui and Chuanxiong doses have a strong nonlinear correlation, and the correlation is positive. When the respective doses of Danggui and Chuanxiong vary greatly, the synergy between the two will increase. When the dose of Danggui increases, the dose of Chuanxiong will also increase significantly.

## 4. Discussion

### 4.1. Descriptive Analysis of Danggui and Chuanxiong Drug Usage

The dosage of Danggui and Chuanxiong is different when treating different indications, but there is an association between the dosage of them when treating the same indications. According to the descriptive statistics of Section 3.1, for the treatment of irregular menstruation, the dose of Chuanxiong was 30 (95% CI: 26.64, 50.44), and the dose of Danggui was 20 (95% CI: 25.56, 61.34). For the treatment of sores, the dose of Chuanxiong was 3.69 (95% CI: 5.48, 13.99), and the dose of Danggui was 3.69 (95% CI: 5.91, 14.72). For the treatment of stroke, the dose of Chuanxiong was 7.38 (95% CI: 16.27, 32.58), and the dose of Danggui was 5.54 (95% CI: 6.02, 33.64). It can be concluded that the dosages of Danggui and Chuanxiong are different in the prescriptions for the treatment of irregular menstruation, sores, and stroke. Besides, the dosages of Danggui and Chuanxiong show related trends when treating the same indications. For example, comparing the prescriptions for the treatment of irregular menstruation, the dosages of Danggui and Chuanxiong are significantly higher than those for the treatment of sores and stroke. Wang examined 1, 242 prescriptions containing Danggui and Chuanxiong drug pairs and reported that, for 68.06% prescriptions, both drugs were prescribed in a 1 : 1 ratio, showing a positive relationship [24]. As it is difficult to draw credible conclusions based only on data description and proportional frequency research, the current study analyzed correlation by use of quantitative statistical methods.

### 4.2. Correlation Analysis of the Dosage of Danggui and Chuanxiong in Different Prescriptions

This study verified the dose dependence of Danggui and Chuanxiong from the perspective of data mining and mathematical statistics by testing the data for normal distribution and calculating the marginal distribution. We found that the dose data for Danggui and Chuanxiong did not conform to the normal distribution and followed a nonlinear and asymmetric trend.

The copula function was first proposed by Sklar [25] as a type of function that connects the joint distribution function with its respective marginal distribution functions, also known as the connection function. This can capture the nonlinearity, asymmetry, and tail correlation between variables [26]. It has been used to explore the dose relationship in clinical medicine. For example, Yin and Yuan proposed a Bayesian adaptive dose discovery design based on the copula model to identify the synergistic effect of dose when two or more drugs are used in combination [27]. Gasparini reported a new type of risk function based on the copula function to evaluate the clinical relevance of combination therapy [28]. However, it has not yet been used in the study of the compatibility of TCM. This study innovatively used the copula function to explore the correlation between the dose changes of Danggui and Chuanxiong. The results of copula function model analysis showed that the doses of Danggui and Chuanxiong are positively correlated in the treatment of irregular menstruation, sores, and stroke, that is, an increase in the dose of one drug increases the dose of the other drug.

### 4.3. Dose of TCM Based on Data Mining and Statistical Research

TCM consists of rich medical theories and clinical prescriptions with curative effects [7, 29]. Analyzing and sorting the dosage of the medicine used by doctors in the past, to study the dosage rule of medicine pairs, can provide more detailed reference and basis of medicine dosage for modern Chinese medicine. It also laid the foundation for the research from quantitative change (dose change) to qualitative change (effect change). The development of data mining technology and mathematical statistics provides an effective technical method for dealing with massive medicines on information resources. This study explored the copula function model through the data mining of the ancient prescriptions, and our results verify the correlation of the drug to the dose. This provides a new research model for the scientific interpretation of the compatibility of traditional classic drugs and may guide the clinical application of TCM in the present world.

## Figures and Tables

**Table 1:** Conversion principles for prescription units in each dynasty.

Dynasty	1 jin (g)	1 liang (g)	1 qian (g)	1 fen (g)
Tang	661	41.31	1.721	0.17
Song	663	40	4.0	0.4
Song	663	40	4.0	0.4
Yuan	663	40	4.0	0.4
Ming	590	36.9	3.69	0.37
Qing	590	36.9	3.69	0.37

**Table 2:** Example of the Danggui and Chuanxiong database.

No.	Prescription name	Dynasty	Danggui dose (g)	Chuanxiong dose (g)	Indications
1	Wenjing Decoction	Song	80	80	Irregular menstruation
2	Foshou San	Song	40	2.6	Sores
3	Tiaojing San	Song	40	20	Irregular menstruation
4	Chaihu Four-Agent Decoction	Jin	60	60	Woman futility
5	Fangfeng Tianmasan	Jin	20	20	Stroke
6	Chaihu Four-Agent Decoction	Jin	4.5	4.5	Postpartum irritability
7	Jiedu Siwutang	Yuan	4	4	Irregular menstruation
8	Jiajian Tenghuang Yinzi	Yuan	3.2	3.2	Sores
9	Shengyanjujing Tang	Yuan	9	3	Irregular menstruation
10	Ejiaosan	Ming	36.9	27.68	Fetal movement
11	Jiawei Wubi Decoction	Ming	3.69	3.69	Paralysis
12	Jiajian Paifeng Decoction	Ming	2.95	2.95	Stroke
13	Hushou Decoction	Qing	36.90	18.45	Headache
14	Supplemented Four-Agent Decoction	Qing	18.45	11.07	Irregular menstruation
15	Jiawei Shengyu Decoction	Qing	11.07	18.45	Sores

Note: we collected 51,083 prescriptions (entire database) based on the recipes from the Han, Tang, Song, Ming, and Qing dynasties. A total of 628 prescriptions containing Danggui and Chuanxiong were identified in the entire database. Table 2 shows 15 sample prescriptions among 628 prescriptions containing Danggui and Chuanxiong.

**Table 3:** Descriptive statistics of variables.

Indications	Variable	Number of cases	M (IQR)	95% CI
Irregular menstruation	Chuanxiong dose	132	30.00 (44.60)	(26.64, 50.44)
	Danggui dose		20.00 (54.00)	(25.96, 61.34)

Sores	Chuanxiong dose	51	3.69 (6.27)	(5.48, 13.99)
	Danggui dose		3.69 (4.00)	(5.91, 14.72)

Stroke	Chuanxiong dose	41	7.38 (38.00)	(16.27, 32.58)
	Danggui dose		5.54 (33.92)	(16.02, 33.64)

**Table 4:** Normality test.

		Kolmogorov–Smirnov			Shapiro–Wilk		
Indications		Variable	d*f*	*P* value	Variable	d*f*	*P* value
Irregular menstruation	Chuanxiong	0.241	216	≤0.001	0.799	216	≤0.001
	Danggui	0.258	216	≤0.001	0.668	216	≤0.001

Sores	Chuanxiong	0.354	57	≤0.001	0.580	57	≤0.001
	Danggui	0.341	57	≤0.001	0.585	57	≤0.001

Stroke	Chuanxiong	0.246	44	≤0.001	0.800	44	≤0.001
	Danggui	0.280	44	≤0.001	0.757	44	≤0.001

**Table 5:** Squared Euclidean distance of the copula function of the irregular menstruation group.

Copula function	Squared Euclidean distance	Parameter estimation
Clayton copula function	0.1024	*α* = 3.4623
Frank copula function	0.0601	*α* = 10.7478
Gumbel copula function	0.0413	*α* = 3.0522
Gaussian copula function	0.0833	*ρ* = 0.0833
*t*-copula function	0.2005	*ρ* = 0.2005; *v* = 8.3468

**Table 6:** Squared Euclidean distance of the copula function of the sores group.

Copula function	Squared Euclidean	Parameter estimation
Clayton copula function	0.0664	*α* = 1.9104
Frank copula function	0.0249	*α* = 7.7570
Gumbel copula function	0.0193	*α* = 2.4568
Gaussian copula function	0.0197	*ρ* = 0.7938
*t*-copula function	0.1240	*ρ* = 0.7137; *v* = 6.3125

**Table 7:** Squared Euclidean distance of the copula function of the stroke group.

Copula function	Squared Euclidean distance	Parameter estimation
Clayton copula function	0.0362	*α* = 6.0456
Frank copula function	0.0116	*α* = 18.3760
Gumbel copula function	0.0099	*α* = 4.4580
Gaussian copula function	0.0353	*ρ* = 0.8914
*t*-copula function	0.0141	*ρ* = 0.9512; *v* = 10.9574

## Data Availability

The data used to support the findings of this study are available from the corresponding author upon reasonable request.

## References

[1] Chen X.-P., Li W., Xiao X.-F., Zhang L.-L., Liu C.-X. (2013). Phytochemical and pharmacological studies on Radix *Angelica sinensis*. *Chinese Journal of Natural Medicines*.

[2] Ran X., Ma L., Peng C., Zhang H., Qin L.-P. (2011). *Ligusticum chuanxiong* Hort: a review of chemistry and pharmacology. *Pharmaceutical Biology*.

[3] Hou Y. Z., Zhao G. R., Yang J., Yuan Y. J., Zhu G. G., Hiltunen R. (2004). Protective effect of *Ligusticum chuanxiong* and *Angelica sinensis* on endothelial cell damage induced by hydrogen peroxide. *Life Sciences*.

[4] Li W., Guo J., Tang Y. (2012). Pharmacokinetic comparison of ferulic acid in normal and blood deficiency rats after oral administration of *Angelica sinensis*, *Ligusticum chuanxiong* and their combination. *International Journal of Molecular Sciences*.

[5] Bi C. W., Xu L., Yu X. (2012). Fo Shou San, an ancient Chinese herbal decoction, protects endothelial function through increasing endothelial nitric oxide synthase activity. *PLoS One*.

[6] Jin Y., Qu C., Tang Y. (2016). Herb pairs containing *Angelicae sinensis* Radix (Danggui): a review of bio-active constituents and compatibility effects. *Journal of Ethnopharmacology*.

[7] Zhang R., Zhu X., Bai H., Ning K. (2019). Network pharmacology databases for traditional Chinese medicine: review and assessment. *Frontiers in Pharmacology*.

[8] Mu R., Xu J. (2017). A new bayesian dose-finding design for drug combination trials. *Statistics in Biopharmaceutical Research*.

[9] Kolev N., Anjos U. d., Mendes B. V. d. M. (2006). Copulas: a review and recent developments. *Stochastic Models*.

[10] Lambert P., Vandenhende F. O. (2002). A copula-based model for multivariate non-normal longitudinal data: analysis of a dose titration safety study on a new antidepressant. *Statistics in Medicine*.

[11] Chen X., Chen J., Cheng G., Gong T. (2020). Topics and trends in artificial intelligence assisted human brain research. *PLoS One*.

[12] Huang J., Lu X.-Q., Zhang C. (2013). Anti-inflammatory ligustilides from *Ligusticum chuanxiong* hort. *Fitoterapia*.

[13] Li W., Tang Y., Chen Y., Duan J.-A. (2012). Advances in the chemical analysis and biological activities of chuanxiong. *Molecules*.

[14] Patton A. J. (2012). A review of copula models for economic time series. *Journal of Multivariate Analysis*.

[15] Li M., Yang N. (2018). Research progress on usual dosage of herbs in traditional Chinese medicine decoction. *Chinese Journal of Experimental Traditional Medical Formulae*.

[16] Wang A. (2019). Research on historical changes of dosage of Chinese medicine based on data analysis. *World Science and Technology/Modernization ofTraditional Chinese Medicine and Materia Medica*.

[17] Pencina M. J., Steyerberg E. W., D’Agostino R. B. (2017). Net reclassification index at event rate: properties and relationships. *Statistics in Medicine*.

[18] Franklin J. M., Rassen J. A., Ackermann D., Bartels D. B., Schneeweiss S. (2014). Metrics for covariate balance in cohort studies of causal effects. *Statistics in Medicine*.

[19] de Leon A. R., Wu B. (2011). Copula-based regression models for a bivariate mixed discrete and continuous outcome. *Statistics in Medicine*.

[20] Neumeyer N., Omelka M., Hudecová Š. (2019). A copula approach for dependence modeling in multivariate nonparametric time series. *Journal of Multivariate Analysis*.

[21] Karra K., Mili L. (2019). Copula index for detecting dependence and monotonicity between stochastic signals. *Information Sciences*.

[22] Chechile R. A. (2005). Matrix analysis for statistics. *Journal of Mathematical Psychology*.

[23] Gretton A. (2012). A kernel two-sample test. *Journal of Machine Learning Research*.

[24] Wang H. (2009). Data analysis of radix *Angelicae sinensis* and *Rhizoma ligusticum* of different proportions in the TCM clinic application. *Chinese Journal of Experimental Traditional Medical Formulae*.

[25] Sklar B. (1997). A primer on turbo code concepts. *IEEE Communications Magazine*.

[26] Thall P. F., Nguyen H. Q. (2012). Adaptive randomization to improve utility-based dose-finding with bivariate ordinal outcomes. *Journal of Biopharmaceutical Statistics*.

[27] Yin G., Yuan Y. (2009). Bayesian dose finding in oncology for drug combinations by copula regression. *Journal of the Royal Statistical Society: Series C (Applied Statistics)*.

[28] Gasparini M. (2013). General classes of multiple binary regression models in dose finding problems for combination therapies. *Applied Statistics*.

[29] Wang S., Hu Y., Tan W. (2012). Compatibility art of traditional Chinese medicine: from the perspective of herb pairs. *Journal of Ethnopharmacology*.

